# Identification of the fungal endophyte of *Ammophila breviligulata* (American beachgrass) as *Epichloë amarillans*

**DOI:** 10.7717/peerj.4300

**Published:** 2018-01-22

**Authors:** Ian Drake, James F. White Jr, Faith C. Belanger

**Affiliations:** 1 Department of Biology, William Paterson University, Wayne, NJ, United States of America; 2 Department of Plant Biology, Rutgers, The State University of New Jersey, New Brunswick, NJ, United States of America

**Keywords:** *Epichloë*, Ammophila, Phylogeny

## Abstract

The grass *Ammophila breviligulata* (American beachgrass) is known to host an endophyte of the genus *Epichloë*. Based on morphological characteristics it was originally identified as *Acremonium typhinum* var. *ammophilae* and is currently designated as *Epichloë typhina* var. *ammophilae*. However, the *Epichloë* species has not previously been identified based on DNA sequence data. Based on phylogenetic placement of beta-tubulin and translation elongation factor 1-alpha DNA sequences the endophyte is identified as a member of *E. amarillans* rather than *E. typhina*.

## Introduction

*Epichloë* spp. (Clavicipitaceae, Ascomycota) are systemic fungal endophytes of many cool season grasses (Schardl et al., 2009; Tadych, Bergen & White Jr, 2014). Infection by these endophytes often provides numerous benefits to the host, such as insect, drought and disease resistance (Clarke et al., 2006; Kuldau & Bacon, 2008). The *Epichloë* endophyte found in some plants of American beachgrass, *Ammophila breviligulata* Fernald (Agrostidinae), was previously designated as *Acremonium typhinum* var. *ammophilae* White et Morgan-Jones, var. nov. (White Jr et al., 1992). The fungal species identification was based on morphological characteristics and was made before the current extensive molecular data on *Epichloë* spp. were available.

The nomenclature of the grass endophytes has since been revised. Based on 18S ribosomal DNA phylogeny, Glenn et al. (1996) proposed that the anamorphic grass fungal endophytes be reclassified from the genus *Acremonium* to the genus *Neotyphodium*. In 2011 the 18th International Botanical Congress ratified a proposal to consolidate anamorphic and teleomorphic fungal species based on the principle of “one fungus = one name” (Norvell, 2011). Leuchtmann et al. (2014) presented a comprehensive review of the known *Epichloë* spp., and proposed a realignment of the anamorphic *Neotyphodium* spp. with *Epichloë*. Based on the previous assignment (White Jr et al., 1992), the species of *Epichloë* infecting *A. breviligulata* was designated as *E. typhina* var. *ammophilae* (J.F. White and Morgan-Jones) J.F. White, comb. nov. (Leuchtmann et al., 2014). However, the *A. breviligulata* endophyte had not yet been subjected to any DNA sequence based analysis. Here we report the phylogenetic placement of the *Epichloë* endophyte of *A. breviligulata* based on beta-tubulin (*tubB*) and translation elongation factor 1-alpha (*tefA*) DNA sequences. In this analysis the endophyte from *A. breviligulata* is placed in a clade with *E. amarillans*, rather than *E. typhina*. We therefore propose that it be considered a member of *E. amarillans*.

## Materials & Methods

### Plant and fungal materials

*Ammophila breviligulata* (American beachgrass) cultivar ‘Cape’ plants were acquired through the USDA Plant Materials Center, Cape May, NJ. The cultivar Cape was developed from a single plant, which was vegetatively propagated, and was released by the Soil Conservation Service, USDA, in 1972 (Gaffney & Duell, 1974). This cultivar is the source of the endophyte previously described by White Jr et al. (1992). The endophyte from all plants of the Cape cultivar therefore originated from a single isolate. Plants were transplanted to pots in standard potting mix for growth in the greenhouse (Pro-mix BX Mycorrhizae, Quakertown, PA), watered to saturation as needed and fertilized weekly with a standard 20:20:20 fertilizer (Plantex 20–20–20 Classic; Master Plant-Prod Inc., Brampton, Ontario). Positive infection status was confirmed by plating surface-sterilized leaf sheath fragments and by microscopic observations, which followed previously published procedures (Bacon & White Jr, 1994; Florea, Schardl & Hollin, 2015).

The fungal endophyte was obtained by surface sterilizing innermost leaf sheath tissue and allowing the fungus to grow out from the host-grass tissue onto potato dextrose agar (PDA) plates. A plate was washed with sterile water and the water spread on a fresh PDA plate to isolate colonies arising from single spores. A single spore colony was isolated and then grown in potato dextrose broth for use in fungal genomic DNA extraction.

### DNA isolation and amplification

Fungal DNA was isolated from 50 mg of tissue by using the Synergy 2.0 Plant DNA Extraction Kit (Ops Diagnostics, Lebanon, NJ, USA). Translation elongation factor 1 alpha (*tefA*) and beta-tubulin (*tubB*) sequences were amplified from the fungal DNA. The primers used for *tubB* gene amplification were tubB-exon1d-1 (5′-GAG AAA ATG CGT GAG ATT GT-3′) and tubB-exon4u-2 (5′GTT TCG TCC GAG TTC TCG AC-3′) and those for *tefA* gene amplification were tefA-exon1d-1 (5′GGG TAA GGA CGA AAA GAC-3′) and tefA-exon5u-1 (5′CGG CAG CGA TAA TCA GGA TAG-3′) (Moon et al., 2002). The 50 µL PCR reactions contained 0.2 µg of fungal genomic DNA, 40 picomoles of each forward and reverse primer (Integrated DNA Technologies, Inc., Coralville, IA, USA), and 25 µl of PrimeSTAR Max Premix (Clontech Laboratories, Mountain View, CA, USA). PCR was performed in a GeneAmp 9700 thermocycler (Applied Biosystems, Inc., Foster City, CA, USA) with 30 cycles of denaturation at 98 °C for 10 s, followed by 15 s annealing at 55 °C, and 2 min extension at 72 °C. The concentration of the PCR product was estimated by running a 5 µl aliquot on a 1% agarose gel and comparing the band intensity with that of the 1000 bp band in the HyperLadder 1kb marker (Bioline USA Inc., Taunton, MA, USA). The PCR products were sequenced directly (Genewiz, Inc., South Plainfield, NJ, USA). For each sequencing reaction, approximately 40 ng of PCR product was treated with 2 µl of ExoSAP-IT (USB Corp., Cleveland, OH, USA) to remove unincorporated primers and excess dNTPs. The ExoSAP-IT reaction was performed at 37 °C for 15 min followed by heating at 80 °C for 15 min to inactivate the enzymes. Sequencing was done in both directions.

### Accession numbers

GenBank accession numbers for the *tefA* and *tubB* sequences are KX523126 and KX523127, respectively.

### Phylogenetic analysis

The *tubB* and *tefA* sequences were aligned with those from non-hybrid *Epichloë* spp. available from the National Center for Biotechnology Information (NCBI; http://www.ncbi.nlm.nih.gov/). The sequences included for comparison are those used previously in the analysis of *E. typhina* subsp. *poae* (see Tadych et al., 2012, Table 1). The Clustal-X program (Thompson et al., 1997) was used to align the sequences, and the alignment was modified manually to minimize gaps. The phylogenetic analysis was performed with the PAUP* program, version 4.0b10 for Macintosh. The phylogenetic analysis was done by using the maximum parsimony full heuristic search option set to random sequence addition, tree-bisection-reconnection (TBR) branch swapping, and Multrees on with 1000 bootstrap replications. Gaps were treated as missing data. The *tubB* tree was based on 469 total characters, of which 377 were constant, 17 variable characters were parsimony uninformative, and 75 variable characters were parsimony informative. The *tefA* tree was based on 804 total characters, of which 582 were constant, 45 variable characters were parsimony uninformative, and 177 variable characters were parsimony informative.

The sequences were also analyzed by the maximum likelihood method in the PAUP* program, which generated trees of similar topology to those of the maximum parsimony analyses (not shown). For the maximum likelihood analyses, the trees were generated with a fast heuristic search using the HKY85 model of sequence evolution, and 100 bootstrap replications.

## Results & Discussion

**Figure 1:**
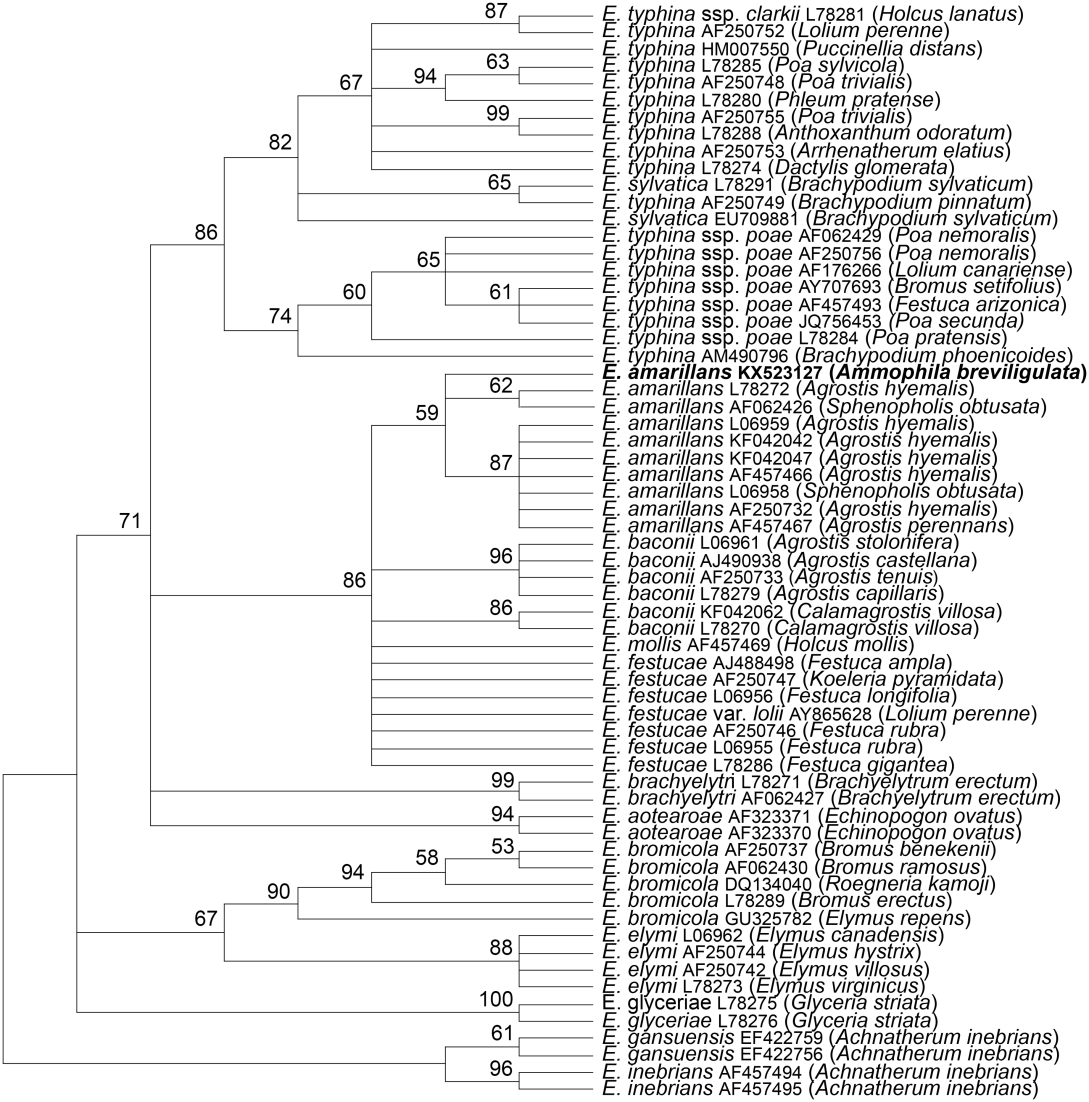
Rooted 50% majority rule consensus maximum parsimony phylogenetic tree of *tubB* sequences. The *E. inebrians* and *E. gansuensis* sequences were designated as outgroups for rooting the tree. The numbers at the nodes are the bootstrap percentages based on 1,000 replications. Sequence references are given by *Epichloë* species names, GenBank accession numbers, and the host grass species are given in parentheses. The *E. amarillans* isolate from *A. breviligulata* is identified by bolded text.

**Figure 2:**
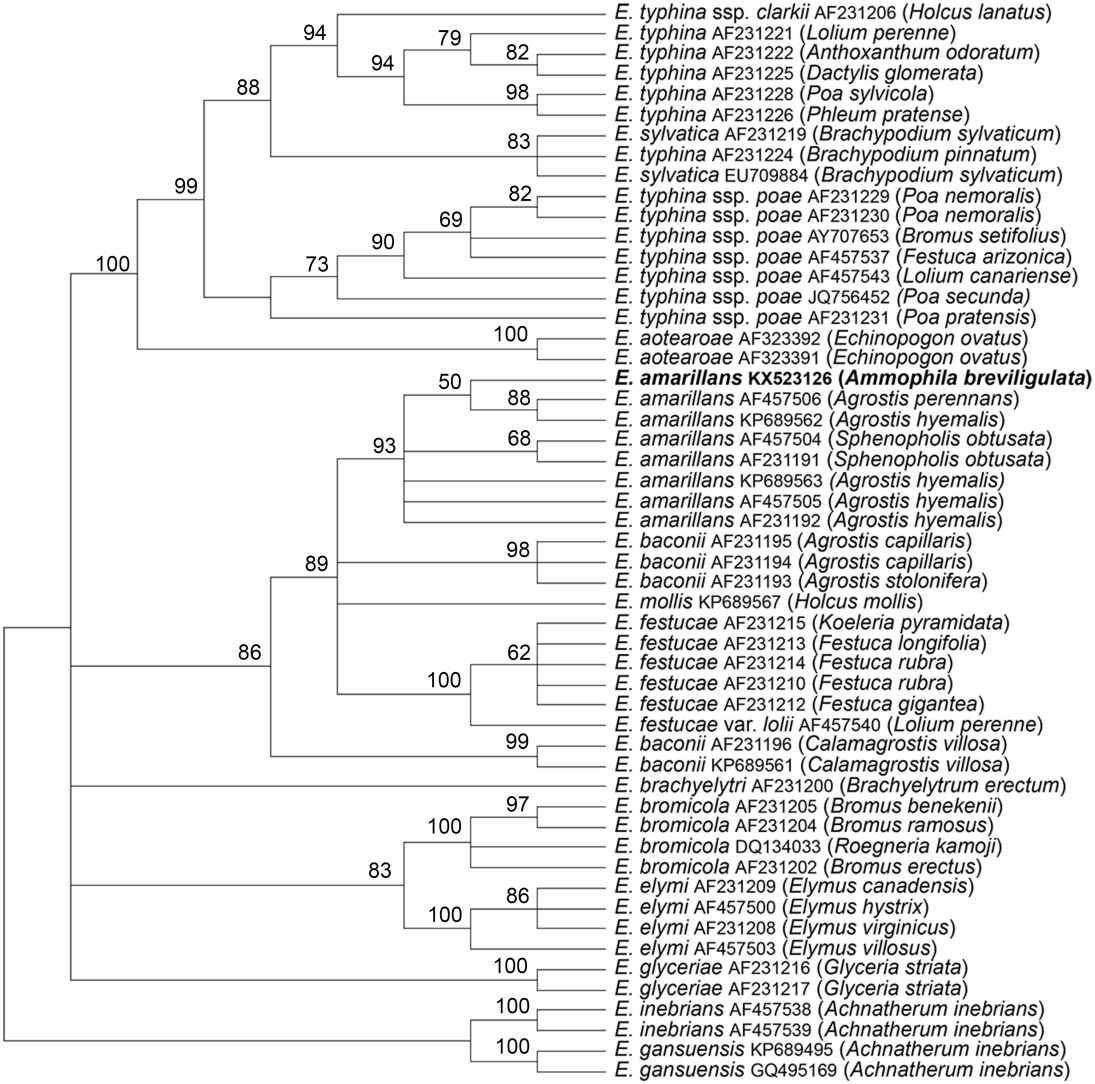
Rooted 50% majority rule consensus maximum parsimony phylogenetic tree of *tefA* sequences. The *E. inebrians* and *E. gansuensis* sequences were designated as outgroups for rooting the tree. The numbers at the nodes are the bootstrap percentages based on 1,000 replications. Sequence references are given by *Epichloë* species names, GenBank accession numbers, and the host grass species are given in parentheses. The *E. amarillans* isolate from *A. breviligulata* is identified by bolded text.

The *tubB* and *tefA* genes were chosen for analysis of the endophyte of *A. breviligulata* since sequences from many *Epichloë* spp. isolates are readily available at NCBI. There was no evidence of heterogeneity that would indicate that the endophyte had multiple gene copies typical for species of hybrid origin. Maximum parsimony phylogenetic analyses of endophyte *tubB* and *tefA* sequences are shown in Figs. 1 and 2, respectively. The species names are those presented in Leuchtmann et al. (2014). The *E. gansuensis* and *E. inebrians* sequences were designated as outgroups for rooting the trees since they are considered the basal *Epichloë* spp. (Ambrose, Koppenhöfer & Belanger, 2014; Chen et al., 2015). In both the *tubB* and *tefA* trees, the sequences from the endophyte of *A. brevilligulata* were placed in the *E. amarillans* clades.

**Figure 3:**
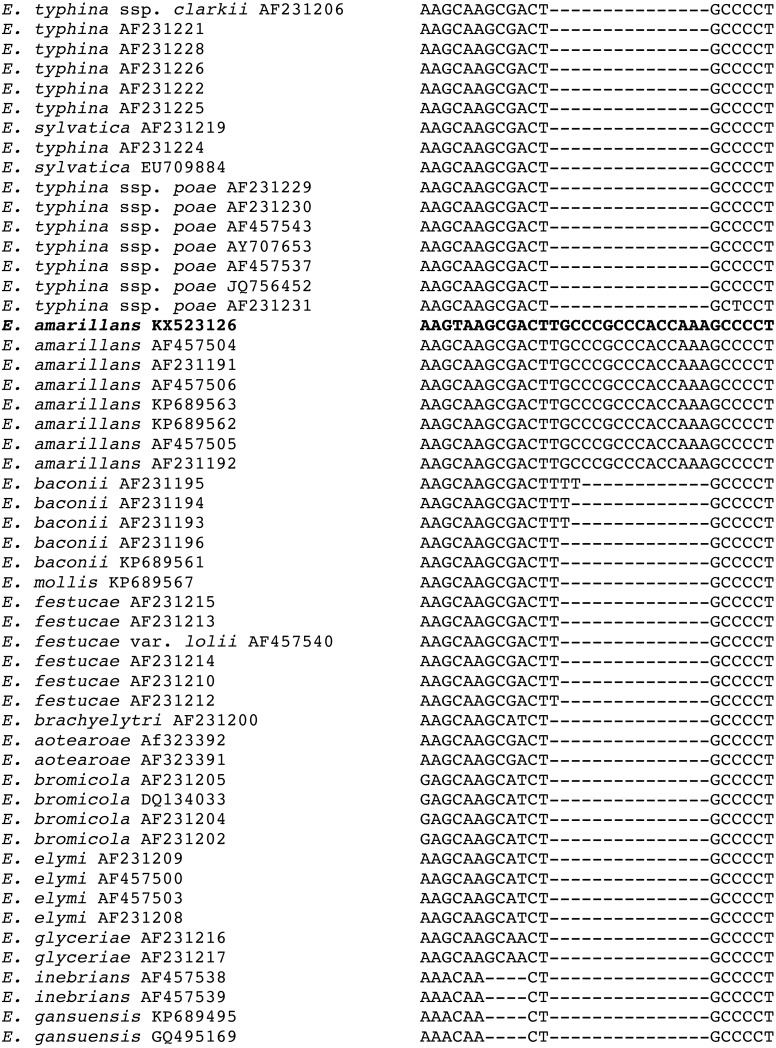
Sequence alignment of the region of the 15 bp insertion in the *tefA* sequences from the *E. amarillans* isolates. The isolate from *A. breviligulata* is identified by bolded text.

Additional support for the species assignment as *E. amarillans* comes from the presence of a shared 15 bp insert in the *tefA* sequence found only in other isolates of *E. amarillans* (Fig. 3). Shared indels are considered to be important phylogenetic characters (Pasko, Ericson & Elzanowski, 2011; Simmons & Ochoterena, 2000; Väli et al., 2008). A shared 19 bp deletion in *tubB* sequences was previously considered as supporting evidence that the *E. typhina* ssp. *poae* isolates infecting several different grass genera had a common progenitor (Tadych et al., 2012).

*A. breviligulata* is ecologically important in shoreline dune building. Endophyte infected *A. breviligulata* was reported to exhibit greater vegetative growth and dune building relative to uninfected plants (Emery, Bell-Dereske & Rudgers, 2015) but was also correlated with reduced species richness at the field site (Rudgers et al., 2015). In these reports the endophyte was referred to as *Epichloë* sp.

In a survey of herbarium samples collected prior to 1971 (White Jr et al., 1992) and a survey of plants collected from natural dunes sites in Michigan and Indiana (Emery, Thompson & Rudgers, 2010), most *A. breviligulata* plants tested were not endophyte infected. However, the cultivar Cape, which is the source of the endophyte analyzed here, is highly infected (White Jr et al., 1992; Emery, Thompson & Rudgers, 2010). This cultivar is commonly used in dune revegetation. Because of the importance of *A. breviligulata* in dune restoration and the widespread dissemination of the endophyte infected cultivar Cape along the East Coast of the United States, it is important to know the taxonomic affiliation of the *Epichloë* endophyte that it is hosting. Based on the phylogenetic data presented here we propose that the endophyte of *A. breviligulata* pertains to the species *E. amarillans*.

## Additional Information and Declarations

### DNA Deposition

The following information was supplied regarding the deposition of DNA sequences: The *tefA* and *tubB* sequences described here are accessible via GenBank accession numbers KX523126 and KX523127.
